# Current Understanding on the Role of Lipids in Macrophages and Associated Diseases

**DOI:** 10.3390/ijms24010589

**Published:** 2022-12-29

**Authors:** Ida Florance, Seenivasan Ramasubbu

**Affiliations:** Centre for Nanobiotechnology, Vellore Institute of Technology, Vellore 632014, Tamil Nadu, India

**Keywords:** lipid droplets, macrophages, microglia, autophagy, hypoxia, HIF-1α, phosphocholines, AMPK

## Abstract

Lipid metabolism is the major intracellular mechanism driving a variety of cellular functions such as energy storage, hormone regulation and cell division. Lipids, being a primary component of the cell membrane, play a pivotal role in the survival of macrophages. Lipids are crucial for a variety of macrophage functions including phagocytosis, energy balance and ageing. However, functions of lipids in macrophages vary based on the site the macrophages are residing at. Lipid-loaded macrophages have recently been emerging as a hallmark for several diseases. This review discusses the significance of lipids in adipose tissue macrophages, tumor-associated macrophages, microglia and peritoneal macrophages. Accumulation of macrophages with impaired lipid metabolism is often characteristically observed in several metabolic disorders. Stress signals differentially regulate lipid metabolism. While conditions such as hypoxia result in accumulation of lipids in macrophages, stress signals such as nutrient deprivation initiate lipolysis and clearance of lipids. Understanding the biology of lipid accumulation in macrophages requires the development of potentially active modulators of lipid metabolism.

## 1. Introduction

ER remains the key regulator of lipid metabolism in a cell. Around one-third of the proteome is synthesized, matured and modified at the rough ER, bound with ribosomes at the membrane. Biosynthesis of lipids, hormone, steroids and xenobiotic detoxification remains the function of smooth ER [1]. The following highlights are the same. The ER houses the enzymes involved in synthesis of cholesterol and triacylglycerides (TAG) [2]. While TAGs are transferred to lipid droplets (LDs) budding from the ER membrane, lipids synthesized at ER are distributed to other organelles via the secretory pathway. At the ER, the cellular cholesterol is controlled via pathways that sense cholesterol levels within the ER membrane and impart signals to control both synthesis and clearance of cholesterol [3]. Under conditions of low ER cholesterol, the primary regulation of cholesterol at the ER involves synthesis of cholesterol via SCAP/SREBP2 (sterol regulatory-element binding proteins 2) pathway [4] followed by conversion of cholesterol into oxysterols and finally into bile acids [5] and production of cholesterol esters which eventually move into lipid droplets [6]. Similarly, ER regulates the intracellular fatty acid composition to regulate the cellular demands required for synthesizing complex lipids. Moreover, ER remains the key regulator of fatty acid synthesis and lipid metabolism. Structural modification of fatty acids such as elongases, desaturases and beta-oxidation cycles occur at ER [7]. SREBPs, a family of membrane bound transcription factors are actively involved in lipid homeostasis. SREBPs, synthesized as precursors reside at the ER membrane [8]. While SREBP1a functions mainly in lipid synthesis in proliferating cells, the major role of SREBP1c remains in regulating the synthesis of triglycerides (TG) and fatty acids in lipogenic organs. SREBP2 widely regulates synthesis of sterols in tissues [9,10]. De novo synthesis of fatty acids is also partially regulated at the ER through feedback inhibition mechanisms of SREBP-1 release [11]. Further, under stimulated TAG synthesis condition, enzymes involved in the biosynthesis of TAG (lipin, DGAT, GPAT and AGPAT) relocalize from ER to lipid droplets [12].

Additionally, synthesis of lipids occurs also at the ER-organelle contact sites. Lipids are trafficked out of ER by specialized ER domains via lipid transfer proteins [2]. It is scientifically plausible that lipid droplets are formed in ER at the regions where synthesis of triacylglycerols (TAGs) or sterol esters takes place [13]. Phospholipids and proteins are also biochemically modified at the ER–Golgi intermediate compartment (ERGIC) and are distributed within the cell via secretory pathways or direct organelle contacts [14,15]. Phospholipids and neutral lipids (TG and CE) are the two primary forms of lipids the ER is comprised of. The key function of phospholipids includes the assembly of membranes and vesicles involved in protein trafficking; TG and CE function as reserves for excess cholesterol and fatty acids and owing to their hydrophobicity, they instigate formation of LDs within the ER membrane [16]. Interestingly, ER -resident DGAT2 (diglyceride acyltransferase) enzyme, pivotal for the synthesis of TG, mediates synthesis and storage of TG in lipid droplets independent of its localization in ER [17]. Further, structurally uniform ER–LD contacts along with the delivery of TGs from ER to LDs were reported to be facilitated by an ER integral protein called seipin [18].

Substantial evidences indicate the role of UPR activation in modulating lipid metabolism by transcriptionally regulating lipogenesis. Moreover, it was reported that ER stress is associated with the imbalance between uptake and efflux of lipids in macrophages [19]. Abnormal lipid metabolism is the key contributor for ER stress [20]. In human hepatic cells, ER stress impaired ATP binding cassette subfamily A member 1 (ABCA1)-mediated cholesterol efflux and negatively affected cholesterol synthesis via down regulation of 3-hydroxy-3-methylglutaryl coenzyme A (HMG-CoA reductase) activity [21]. Further, the similarity in the processes involved in the activation of ATF6 and SREBPs imply the role of ER stress in lipid metabolism [22,23]. In addition, prolonged ER stress dysregulates hepatic lipid metabolism. In contrast, excess cholesterol and saturated fatty acids induce ER stress [24,25]. Such lipid imbalance caused by ER stress directs to lipotoxicity-related pathologies. Furthermore, ER stress is known to increase the expression of SREBP-dependent markers of lipogenesis via UPR [26].

**Figure 1:**
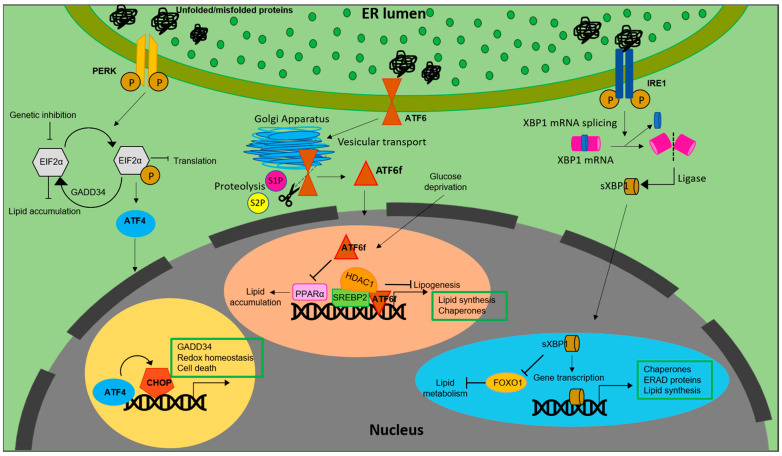
Lipid biosynthesis at ER. The schematic representation shows the mechanism of lipid biosynthesis taking place at the ER via the three branches of the UPR: ATF6, PERK and IRE1.

It is also notable that the PERK-eIF2α pathway regulates lipogenesis. For example, antipsychotic drugs induced phosphorylation of PERK and eIF2α, resulting in SREBP-1c and SREBP2 mediated accumulation of lipids in hepatic cells [27] (Figure 1).

Similarly, inhibiting eIF2α phosphorylation via overexpression of growth arrest and DNA damage-inducible gene 34 (GADD34) in liver decreased the hepatosteatosis in mice fed with a high fat diet (HFD) [28]. Genetic deletion of eIF2α aggravated tunicamycin-induced accumulation of lipids in liver [29]. ER stress inducers, brefeldin A and tunicamycin, were reported to induce LD accumulation in Saccharomyces cerevisiae [30]. Further, ATF4, present downstream of the PERK-eIF2α pathway, plays a pivotal role in regulation of lipid metabolism. High carbohydrate diet fed Atf^−/−^ mice displayed less accumulation of TG in liver compared to wild type [31]. Correspondingly, mice lacking ATF4 showed diminished lipid accumulation under conditions of high fructose diet due to decreased levels of FAS, acetyl CoA carboxylase (ACC) and SREBP-1c [32]. White adipose tissue of Atf deficient mice displayed increased lipolysis and decreased lipogenic genes, indicating a direct link between ATF4 and lipid metabolism [33]. Similarly, overexpressing ATF4 instigated early onset of dyslipidemia in zebrafish [34]. The involvement of (C/EBP) homologous protein (CHOP), a protein downstream of the UPR, in the regulation of lipid metabolism emanates from its role in suppressing the gene expression of SREBPF1, CEBPA, and PPARα-like master regulators of lipid metabolism [35]. Following ER stress, CHOP was reported to be critical for the regulation of cholesterol catabolism in macrophages and is involved in the lipid metabolism disorder mediated by ER stress [19]. In mammary epithelium and mouse embryonic fibroblasts that differentiate into adipocytes, absence of PERK resulted in the attenuation of lipogenesis and expression of genes including SREBP1. This led to a decrease in the level of TG and FA content in mammary glands and growth retardation in pups. The study also demonstrated the role of PERK and eIF2α in insig1 translation responsible for SREBP1 activation [36].

Another transmembrane signal transducer of the UPR, the inositol requiring enzyme 1 (IRE1α), is an ER stress sensor, conserved from yeast to mammals [37,38]. During ER stress, IRE1α is activated and splices X-box-binding protein 1 (XBP1) mRNA to its active spliced form to regulate expression of genes involved in restoration of ER homeostasis and biogenesis [39]. In addition to promoting cell survival through attenuation of ER stress, IRE1α also functions as a nutritional stress sensor [40]. Further, IRE1α was found to regulate lipid secretion and lipogenesis in both XBP1-dependent and an independent manner [41,42] (Figure 1). In c-Myc-overexpressing and IRE1α inhibited BL cells, defects in growth and viability were triggered due to altered lipid homeostasis [43]. The IRE1a/XBP1 signaling pathway transcriptionally regulates genes that are players of lipid metabolism in order to activate hepatic lipid metabolism. IRE1α siRNA increased TG and cholesterol levels in XBP1-deficient mice. This suggests that IRE1α hyperactivation reduced plasma lipids under XBP1-deficient conditions. Ablation of XBP1 decreased hepatoxicity [44]. However, how IRE1α functions to maintain lipid homeostasis in peripheral adipose tissues across species remains an enigma. As reviewed by Basseri et al., accumulating evidences suggest that IRE1α is the key component involved in the suppression of hepatic lipid accumulation under conditions of severe ER stress [45]. Under ER stress, IRE1α plays a pivotal role in hepatocytic secretion of LDL and VLDL [42]. XBP1s reduces lipid accumulation by promoting protein degradation of Forkhead box protein O1 (FOXO1) in cardiomyocytes. Similarly, overexpressing XBP1s specifically in cardiomyocytes, mitigated cardiac steatosis [46].

In contrast to IRE1α and PERK, activation of ATF6 does not involve phosphorylation. Under conditions of ER stress, ATF6 is released from BiP, after which the Golgi-localization sequences on the luminal domain of ATF6 are exposed [47] Once transported to Golgi, ATF6 undergoes site-1 (S1P) and site-2 (S2P) proteases mediated cleavage, releasing a cytosolic fragment called ATF6f containing a basic leucine zipper (bZIP) transcription factor [22,48]. The transcriptionally active ATF6 fragment enters the nucleus to trigger a set of transcriptional signaling, to reestablish the ER homeostasis [49,50,51]. Unlike PERK and IRE1 branches of UPR, ATF6 does not function to decrease the flux of unfolded proteins into the ER. Instead, the active fragment induces expansion of the ER membrane in an XBP1-independent manner [52] It also upregulates ER chaperones, ERAD components and disulfide oxidoreductases of the ER lumen [53]. However, ATF6 and XBP1 generated from the IRE1 branch of UPR tend to act synergistically and heterodimerize [54]. ATF6 activation downregulates PPARα, leading to accumulation of lipid droplets resulting in cell death. In contrast, deficiency of ATF6 increased PPARα levels and decreased lipid accumulation and cell death [55]. ATF6 was reported to modulate SREBP2 mediated lipogenesis. Under glucose-deprived conditions, ATF6 interacts with the processed form of SREBP2 to inhibit cholesterol synthesis promoting recruitment of HDAC1. This inhibits the SREBP2-induced lipogenesis and downregulates LDLR expression in HepG2 cells [56]. Liver-specific over expression of ATF6f resulted in the improvement of hepatic condition in steatosis-induced mice fed with a high-fat high-sucrose diet. However, ATF6 was reported to not affect FA synthesis, suggesting that ATF6 activity in liver is more important for FA oxidation than synthesis [57]. This is consistent with the finding that ATF6 expression aids protection against perturbed beta oxidation induced by tunicamycin in liver but not in the kidneys [58].

## 2. Functions of Lipids in Macrophages of Different Tissue Location

### 2.1. Lipids and Microglia

Microglia are specialized immune cells resident in the central nervous system (CNS) of the brain and play a key role in the maintenance of brain homeostasis [59,60,61]. Originating from the yolk sac, they migrate to the CNS during embryogenesis; there they propagate and disperse in the CNS in a non-heterogenous manner [62,63]. In a healthy resting brain, microglia are reported to be dynamic and constantly moving [64]. They critically survey the brain environment and get activated upon changes in the brain microenvironment [65]. Their functions include phagocytosis of apoptotic bodies and debris, neuronal protection [64], synaptic remodeling [66,67], neuronal support [68,69] and oligodendrogenesis [70,71]. Microglia dysfunction is a salient feature in neurodegenerative and neuroinflammatory diseases. They become dysfunctional with aging; dysfunctionalities include poor cholesterol efflux, impaired phagocytosis and increased secretion of cytokines and accumulation of lipid droplets [59,72]. Dysregulated lipid metabolism is a characteristic feature observed in neurodegenerative diseases, notably, in Parkinson’s disease (PD) and Alzheimer’s disease (AD). Lipid metabolism in microglia is tightly regulated both during development and disease. Microglia play an inevitable role in maintaining the myelin dynamics. During early development, microglia phagocytose myelin debris and apoptotic oligodendrocytes. The myelin-derived lipids are then cleared by microglia for remyelination post demyelination. Such active clearance and ability to effectively efflux cholesterol is impaired in aging microglia [73,74]. This leads to accumulation and crystallization of cholesterol-rich myelin debris, resulting in defective phagocytosis. Microglia with defective phagocytosis tend to produce large amounts of pro-inflammatory cytokines and reactive oxygen species (ROS) leading to the progression of neuroinflammatory and neurodegenerative diseases [75,76,77]. In a recent study, Loving et al., elucidated the role of lipoprotein lipase (LPL) in the accumulation of LDs and transcriptional regulation of lipid metabolism in in vitro and ex vivo systems. They reported that LPL regulates lipid metabolism in microglia and that loss of LPL resulted in microglial cholesterol load [72]. Phagocytosis deficit can be the consequence of lipid droplets accumulation. Active degradation of lipid droplets and release of free fatty acids was associated with effective phagocytosis [78].

Similarly, lipid droplet accumulating macrophages (LAMs) show downregulated expression of two key enzymes ADRB1 and ADRB2, involved in lipid degradation [59]. Additionally, lipid droplet accumulation promotes transcriptional modulation, giving LAMs a unique transcriptomic signature. Further, Patel and Tulsi et al. reported the association of pathways of lipid and carbohydrate metabolism with sex, age and ApoE expression in human microglia [79]. ApoE is a protein that mediates metabolism and transportation of cholesterol and is prominently expressed in disease-associated microglia (DAM). Moreover, it was reported that extracellular ApoE can be a ligand of TREM2 (triggering receptor expressed on myeloid cells 2) and that ApoE expression is TREM2-dependent [80,81]. Analysis of cell-specific lipidomics reveal that TREM2 deficiency mediates dysregulation of genes associated with lipid metabolism and leads to cholesterol ester overload in microglia [82]. Understanding the link between lipids and neuropathology of NPC (Niemann–Pick disease) patients revealed that loss of NPC1, an intracellular cholesterol transporter in microglia resulted in enhanced uptake of myelin but impaired myelin turnover. Macrophages derived from the blood of NPC patients were found to be similar to the pathological alterations exhibited by microglia of Npc1^−/−^ mice. It was also revealed that Npc1^−/−^ deficient microglia accumulated undigested lipid materials, indicating the role of Npc1 in lipid trafficking in microglia [83].

### 2.2. Lipids in Adipose Tissue Macrophages (ATMs)

Initially discovered for their role in microbial killing and phagocytosis, macrophages are now known to have distinct and context-dependent functions in different physiological settings. Lipids are the major source of energy for macrophages. Cell membranes of macrophages and precursors of bioactive lipids are provided by lipids. Lipids are also known to regulate the signal transduction during macrophage activation. Activation or polarization of macrophages are dependent on environmental stimuli, which are even tissue-specific, that dictate them to take up unique functions.

Adipose tissue macrophages (ATMs) are key players in metabolic diseases and obesity-associated inflammation. Circulating monocytes accumulating in adipose tissue lead to the development of ATMs [84]. In a study conducted by Prieur et al. in obese mice, it was observed that increase in the accumulation of lipids in ATMs resulted in polarization of macrophages into M1 phenotype, a phenotype associated with insulin resistance and obesity. Their results indicate that M1 polarization of ATMs are associated with accumulation and proliferation of lipid species, giving them the resemblance of vascular foam cells (discussed later). In addition to M1 polarization, ATMs of obese mice strongly accumulated lipids in their cytoplasm, resembling pro-atherosclerotic vascular foam cells. Increased fat deposition in adipose tissue decreased the expandability of adipose tissue, leading to adipocyte dysfunction and lipid leakage and thereafter lipid accumulation in ATMs [85]. Similarly, M1 macrophages treated with exogenous fatty acids showed an increase in TG and CE levels. The accumulation of exogenous fatty acids was high in M2 macrophages, revealing the impact of macrophage polarization on lipid composition and endogenous lipid pools [86]. ATMs of obese individuals crucially function to scavenge and eliminate adipocyte debris. Under increasing conditions of adiposity, excess lipid species are stored in ATMs, leading to the formation of lipid-laden ATM population [87]. Cd36, Fabp4, Fabp5 and Lpl are the set of genes that are highly conserved in lipid-associated ATMs [88].

Lipid-associated macrophages (LAMs) are distinctly conserved subset of macrophages predominantly expanded in adipose tissues of obese individuals. The formation of LAMs in adipose tissues are driven by a TF called TREM2. TREM2-regulated TAMs were reported to be inevitable during the loss of adipose tissue homeostasis, as they prevent metabolic disorders [88]. Suppression of tumor growth of triple-negative breast cancer (TNBC) was achieved by genetically depleting LAM subsets [89].

Atherosclerosis is another condition where the lipid homeostasis of macrophages is disrupted. During atherosclerosis, initially, the modified lipoproteins and serum lipids are deposited under the endothelial cells, activating them to secrete adhesion molecules. Circulating monocytes interact with these adhesion molecules that adhere to the endothelial cells and migrate to subendothelial space where they are differentiated into macrophages. Ingestion of modified lipoproteins by macrophages takes place via receptor mediated phagocytosis or pinocytosis. As a result of excess uptake of lipids, macrophages tend to store the excess neutral lipids in the form of lipid droplets in the cytoplasm. Excess accumulation of lipid droplets (LDs) in macrophages gives them a foamy appearance and hence they are called “macrophage foam cells”. These lipid-laden macrophages are the hallmark of atherosclerosis.

Formation of lipid-laden macrophage foam cells in lungs occur even during the infection of *Mycobacterium tuberculosis*. In response to TB infection, macrophages undergo metabolic changes and develop into foam cells. Though they resemble atherosclerotic foam cells, their lipid composition and roles remain different. Unlike atherosclerotic foam cells, their lipid content is predominantly triglycerides (TG) and not cholesterol. Here, the formation of TB foam cells is attributed to mycolic acid from pathogens. These TB foam cells often dominate the mycobacterial granulomata associated with caseum [90].

### 2.3. Lipids in Tumor Associated Macrophages (TAMs)

TAMs are macrophages present in the microenvironment of solid tumors, creating an immunosuppressive environment. Lipids play a key role in the development of TAMs in the tumor microenvironment (TME). Accumulating evidence suggest that abnormal lipid accumulation is inevitable for TAMs to engage in protumorigenic activity. Tumorigenesis and tumor progression is associated with the functional plasticity of TAMs which is often dictated by their metabolic features. The metabolic and functional landscape of tumor cells keeps evolving according to the selective pressure of the inconsistency in the availability of nutrients and oxygen in the TME, as a result of which functional features of TAMs are often altered [91]. Macrophages from both murine and human tumors were found to express high levels of a scavenger receptor, CD36, and ingest more lipids [92] for use as source of energy via oxidative phosphorylation and fatty acid oxidation. Contradictorily, enhanced fatty acid oxidation in macrophages caused by fatty acids in tumor microenvironment results in increased ROS production and decreased IL-10 secretion to eliminate tumor cells. This signifies the involvement of lipid metabolism in anti-tumor response [93]. Similarly, in prostatic adenocarcinoma (PCa), TAMs in the TME were shown to have dysregulated lipid metabolism. Accumulation of lipids in TAMs was reported to positively correlate with the progression of PCa [94]. TAMs characterized as M2-like cells, suppress tumor immune surveillance to promote tumor growth and metastasis. In a study, Wu et.al., demonstrated that enhancing lipid metabolism is sufficient for modulating the phenotype of macrophages into immunosuppressive TAMs [95]. Further, ingestion of a high amount of lipids from tumor cells leads to over expression of phosphoinositide 3-kinase (PI3K-γ) resulting in the polarization of TAMs into an M2-like phenotype. Inhibiting PI3K-γ reversed the pro-tumor phenotype of LD-loaded TAMs, suppressing the growth of gastric cancer [96]. In a study conducted on a melanoma model, it was reported that β-glucosylceramide released by tumor cells served as a stimulus for protumorigenic polarization of TAMs via induction of ER stress responses-mediated shuffling of lipid composition in macrophages [97]. In addition to lipid accumulation, TAMs exhibit decreased phagocytic activity with upregulated expression of programmed death ligand 1 (PD-L1); they block anti–tumor T cell responses to support immunosuppression [96]. From these reports, it is scientifically evident that, lipid droplets are critical cell structures that can be targeted for the development of a novel anti-tumor strategy and that reprogramming lipid metabolism can maximize the impact of anti-tumor therapies.

### 2.4. Lipids in Phagocytic Function of Macrophages

Macrophages are phagocytic in nature. Through phagocytosis, macrophages engulf foreign organisms and other invading pathogens, thereby defending the host against infection. The crosstalk between hypoxia and inflammation has a significant implication for infection and sterile inflammation in macrophages. Macrophages are the primary component of the innate immune response that is known for phagocytosis of invading pathogens and microorganism. Apoptotic cells are also eliminated by macrophages via phagocytosis. Formation of lipid-rich organelles, called lipid bodies or lipid droplets (LDs), occur in parallel with formation and maturation of phagosomes containing pathogens [98,99]. Infections with microbes such as bacteria, virus and other parasites induced LD accumulation in immune cells both clinically and experimentally [100,101]. These lipid bodies, formed in response to infections relocate within cytoplasm to interact with the phagosomes [102]. However, this association between LDs and phagosomes is yet ill-understood. Nevertheless, this interaction is accounted for the survival of pathogens within host cells.

### 2.5. Oxidized Phosphocholines in the Immune Function of Macrophages

**Figure 2:**
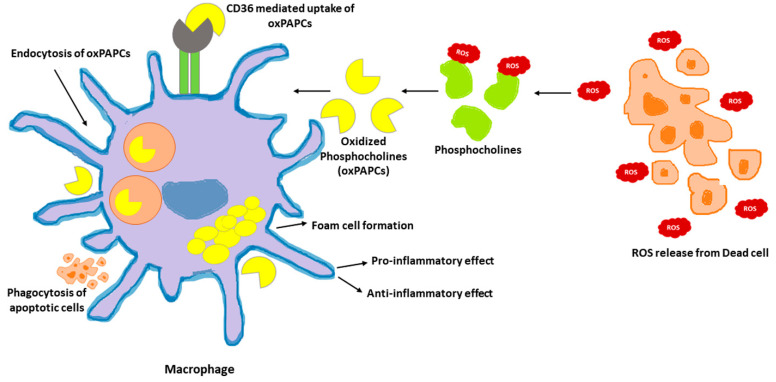
oxPAPCs in the regulation of macrophage immune function. Oxidized by the reactive oxygen speices (ROS) released from dead cells, phosphocholines are taken up by macrophages via endocytosis or CD36 scavenging receptors. Internalized oxPAPCs lead to the formation of foam cells by causing lipid accumulation in macrophages. The oxPAPCs accumulated macrophages are both pro- and anti-inflammatory based on the context.

Phosphocholines, belonging to the class of phospholipids, are a vital component of mammalian cells. Cell death occurring in the local environment of inflammatory or non-inflammatory tissue injury results in ROS generation. This can oxidize the phosphocholines present in the plasma membrane. Upon exposure to ROS, arachidonic acid-containing phospholipid 1-palmitoyl-2-arachidonoyl-sn-glycero-3-phosphocholine (PAPC), the membrane component of mammalian cells, is oxidized at different positions, creating a heterogenous mixture of lipids called oxPAPCs. The scavenger receptor CD36 present on macrophages, induce the uptake of oxPAPCs. This leads to formation of foam cells during atherosclerosis [103]. The internalization of oxPAPCs into endosomes and their transport to cytosol is also mediated by bacterial lipopolysaccharide (LPS) receptor, CD14 present on myeloid cells [104]. Post recognition by macrophages, oxPAPCs exert proinflammatory or anti-inflammatory activities based on the context in which they were encountered [105]. Pre-treatment with oxPAPCs modulated the phagocytosis of bacteria in macrophages [106]. In contrast, oxPLs trigger CD36-mediated phagocytosis of apoptotic cells in macrophages [107]. Further, oxPAPCs perform a metabolic rewiring in macrophages which is speculated to increase mitochondrial fitness. This, in turn, could prolong their lifespan [108]. In addition, pure lipids present in oxPAPCs contribute to metabolic hyperinflammation. In addition, oxPAPCs are stimulators of inflammation in airways. During lung injury, oxPAPCs promote secretion of IL-6 from alveolar macrophages [109]. In contrast, pre-treatment of macrophages with oxPAPCs was reported to block the response of nuclear factor-κB to LPS treatments. The competitive interaction of LPS and oxPAPCs with CD14 remains the underlying mechanism [110]. The mechanism of oxPAPCs mediated accumulation of lipids in macrophages is shown in Figure 2.

## 3. Modulators of Lipid Metabolism at a Glance

Several modulators of lipid metabolism have been identified. Of these, lipid lowering compounds are of high interest as most of the diseases associated with lipid metabolism are characterized by increased levels of lipids and cholesterol. IFNγ stimulates lipid accumulation via upregulation of ACAT-1 mRNA expression [111]. Further, adipophilin promotes triglyceride and cholesterol storage leading to increased cholesterol accumulation and reduced efflux [112]. Further, YC-1, a potential anticancer drug induces lipid accumulation via the sGC/cGMP/PKG pathway [113]. Torcetrapib is a lipid lowering drug that functions by inhibiting cholesterol ester transfer protein (CETP). However, the drug has been postulated to exert adverse off-target effects such as increased aldosterone levels in plasma and blood pressure. The adverse outcome of the drug poses questions on whether CETB inhibition or the molecule itself is the reason behind the failure of torcetrapib [114]. Statins are another group of lipid-lowering drugs that lower TG, LDL and VLDL levels via inhibition of HMG CoA reductase. Fibrates are a class of lipid lowering drugs that decrease TG and LDL levels and increase HDL cholesterol levels. Gene therapies are also well-established to lower lipids. Further, these drugs are also used in combination to achieve a maximum decrease in cholesterol levels. Additionally, key transcription factors involved in the integration of autophagy in lipid metabolism are potential targets for modulating lipid metabolism. Autophagy inducers can effectively lower lipids. For example, the transcriptional factor AMPK is involved in the regulation of four major mechanisms: lipid metabolism, glucose metabolism, autophagy and mitochondrial homeostasis [115]. Such key players can evidently be targeted for repurposing as modulators of lipid metabolism. Table 1 lists the modulators of lipid metabolism and their mechanism of action.

## 4. The Network of Lipids and ROS in Macrophages

Accumulating studies have implied the putative role of ROS in regulating lipid metabolism in different cell types. The role of ROS in cellular processes varies for cell types; while non-phagocytic cells utilize ROS for various functions such as gene expression, signal transduction and other physiological roles, phagocytic cells such as macrophages utilize ROS for killing invading pathogens. ROS generation plays an important role in macrophage polarization. Cellular events such as uptake and oxidation of fatty acids during the activation of M2 macrophages infer the key role of lipid metabolism in macrophage polarization. ROS mediated the Lipopolysaccharide (LPS)-induced differentiation of THP-1 monocytes and activated HIF-1α [142]. Similarly, elimination of ROS using ROS inhibitors resulted in complete blockage of monocyte/macrophage differentiation [143]. In addition, ROS-mediated oxidation of lipids and proteins presented on membranes of apoptotic cells facilitated the recognition and attachment of macrophages to the dying cells [144]. Moreover, macrophages failed to identify and engulf apoptotic cells when the oxLDL epiptopes on the apoptotic cells were blocked using mitochondrial antibody [145], indicating the crucial role of ROS in regulation of lipid metabolism for the phagocytic activity of macrophages. The functions of ROS and lipid metabolism are interchanging in macrophages; resolvin D1 (RvD1), a docosahexaenoic acid derivative, a specialized endogenous pro-resolving lipid mediator, functions to inhibit ROS generation from apoptotic cells after their encounter and engulfment by macrophages, thereby preventing excess ROS-mediated macrophage cell death [146].

## 5. HIF-1α, a Master Regulator of Lipid Metabolism

HIF-1α is a metabolic regulator, that participates in M1 polarization of macrophages. Overexpression of HIF-1α increased the expression of genes involved in glycolysis. This eventually mediated the M1 polarization of macrophages [147]. This function of HIF-1α in activating inflammatory macrophages signifies its role in the crosstalk of inflammation and hypoxia in macrophages. Contrastingly, activation of HIF-1 was reported to attenuate periapical inflammation. HIF-1α activation by dimethyloxalylglycine (DMOG) specifically inhibits the LPS-induced M1 polarization of macrophages while increasing the M2 polarization. Further, this also resulted in the suppression of NF-κB and proinflammatory cytokine production in macrophages [148]. In addition, the role of HIF-1α as a metabolic regulator in the immune function of macrophages is confirmed by the fact that HIF-1α-mediated glycolysis was reported to be essential for the function of pro-inflammatory macrophages to protect against fungal and bacterial infections [149].

Tissue-resident alveolar macrophages (TR-AMs) are a population of macrophages that are functionally distinct and are often characterized by low glucose and high oxygen. The effector function of TR-AMs is predominantly reliant on mitochondrial function rather than glycolysis. Progressive loss of TR-AMs is observed in acute respiratory distress syndrome (ARDS). Hypoxia was reported to stabilize HIF-1α promoting glycolytic phenotype in TR-AMs, resulting in the attenuation of acute lung injury in mice [150]. Expression levels of HIF have been shown to be upregulated by ligands of Toll-like receptor 4 (TLR-4) similar to LPS in macrophages. Under hypoxic conditions, protein synthesis of HIF-1α in human peripheral T cells increased with T-cell receptor (TCR) engagement [151].

**Figure 3:**
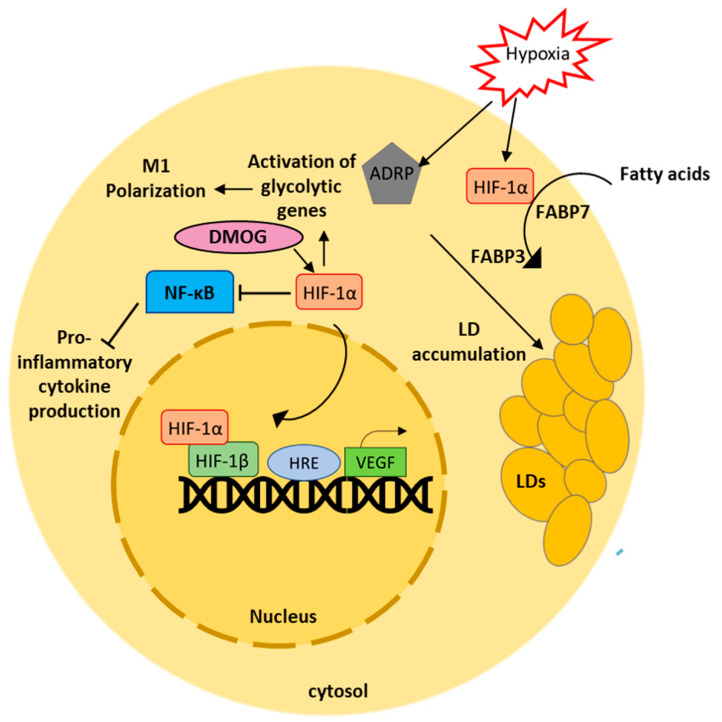
Hypoxia induces HIF-1α mediated lipid accumulation in macrophages. Activation of HIF-1α induces lipid accumulation in macrophages via activation of ADRP. Uptake of fatty acids in macrophages is facilitated by HIF-1α. DMOG mediated activation of HIF-1α suppresses production of pro-inflammatory cytokines. HIF-1α mediates macrophage polarization by activating genes involved in glycolysis.

During atherosclerosis, the arterial walls tend to thicken, exceeding the diffusion limit of oxygen. In atherosclerotic plaques, the hypoxic regions are usually filled with lipid-laden macrophage foam cells. Hypoxia-inducible factor-1 (HIF-1) is a transcriptional factor that plays a key role in the process of adaptation to hypoxia. During hypoxia, the HIF-1α subunit of HIF-1 undergoes nuclear translocation to heterodimerize with HIF-1β [152]. Post-translocation, HIFs bind to hypoxia-response elements (HREs) or to vascular endothelial growth factor (VEGF) such as oxygen-sensitive genes. HIF-1α was reported to affect lipid metabolism in cancer cell lines. Hypoxia-induced lipid accumulation has been found to decrease in macrophage-like cells lacking HIF-1α [153]. Expression of HIF-1α promotes the development and progression of atherosclerosis. Under normoxic conditions, expression of HIF-1α is upregulated in activated macrophages. In atherosclerotic plaques, they are associated with an atheromatous inflammatory plaque phenotype [154]. Accumulation of lipid droplets (LDs) during hypoxia is due to the activation of adipophilin (ADRP) which is crucial for uptake of FA via activation of FABP3 and FABP7 instead of de novo FA synthesis and for the formation of LD membranes. In tumor cells, hypoxia induces accumulation of LDs in HIF-1α dependent manner. Inhibition of HIF-1α expression resulted in decreased levels of LDs after hypoxia [155]. Additionally, in apolipoprotein E deficient mice, accumulation of neutral lipids in macrophages that contributes to atherosclerosis was mediated by hypoxia-inducible protein 2 Hig2/Hilpda [156]. Exposure of human macrophages to hypoxic conditions resulted in the cytosolic accumulation of TG-containing LDs [157]. The mechanism of hypoxia-mediated lipid accumulation is shown in Figure 3. Nevertheless, the cellular response to hypoxia differs from cell to cell and is often prone to modulation by several environmental cues.

## 6. Integration of Autophagy in Lipid Metabolism

Autophagy is an intracellular, self-degradative mechanism that balances energy sources and plays a housekeeping role, in that it eliminates aggregated misfolded proteins and clears damaged organelles. Moreover, autophagy plays a key role as a defense mechanism during conditions of defective lipid metabolism. Autophagy is a molecular switch to regulate lipid metabolism via activation of lipolysis. In contrast, autophagy is also reported to contribute in the formation of lipid droplets by mechanisms still unclear [158]. Certain transcriptional factors regulate lipid metabolism in cells by integrating autophagy. Their interaction with transcriptional regulators of various stress responses dictates the cells to switch directly between pathways.

### 6.1. PPARα

**Figure 4:**
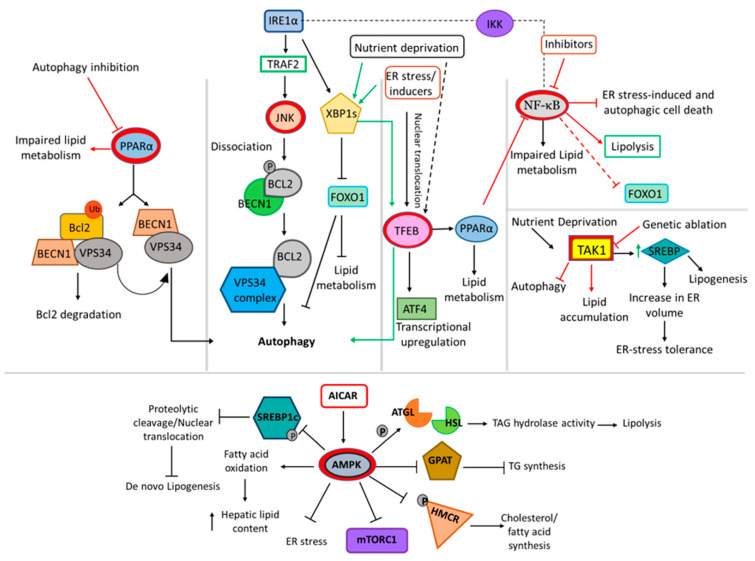
Integration of autophagy in lipid metabolism by selective key players. The illustration shows the molecular network of key transcriptional factors involved in connecting autophagy and lipid metabolism. PPARα, JNK, TFEB, NF-Κb and AMPK are the major transcriptional factors involved in the regulation of autophagy and lipid metabolism among several other pathways.

Primarily, expressed in the heart, kidney, brown adipose tissue, PPARα is involved in the expression of genes related to fatty acid transport [159]. Activation of PPARα effectively eliminates lipotoxicity [160]. Supporting the role of PPARα, extended in the linkage, autophagy-deficient cells displayed impaired lipid oxidation and decreased PPARα protein expression [161]. PPARα-autophagy functions to promote cell survival [162]. PPARα transcriptionally activates β-oxidation upon sensing the fatty acids. This process involves the activation of a key player of autophagy, called AMP-dependent protein kinase (AMPK), which functions to sense the availability of AMP/ATP to initiate pro-survival autophagy (Figure 4). It was also reported that pharmacological inhibition of autophagy with chloroquine (CQ) caused accumulation of LDs and cellular TG content with decreased PPARα protein levels in zebrafish liver cells [163]. This clearly indicates that PPARα regulates the non-autophagic function of AMPK [164].

### 6.2. JNK

Another key player in the axis is c-Jun N-terminal kinase (JNK). Activated IRE1α phosphorylates JNK which in turn phosphorylates Bcl2, dissociating it from BECN1 to induce autophagy. JNK plays a pivotal role in the autophagy–lipid metabolism axis (Figure 4). Hepatic deficiency of JNK resulted in dysregulation of lipid homeostasis [165]. In contrast, inhibiting JNK activation resulted in ameliorated insulin signaling in adipocytes treated with FFA [166]. Correspondingly, both genetic and pharmacological inhibition of JNK alleviated saturated FFA-mediated lipopaoptosis in hepatocytes. Moreover, autophagic cell deaths had been reported to be mediated by JNK [167].

### 6.3. AMPK

Under normal physiological conditions, AMPK remains inactive while mTORC1 remains active. Active mTORC1 then participates in the synthesis of lipids via PPARγ and SERBP1c. AMPK directly phosphorylates SREBP1c to inhibit its proteolytic cleavage and nuclear translocation, thereby reducing de novo lipogenesis [168]. In addition, mTORC1 inhibits β-oxidation of fatty acids by inactivating PPARα. The overall lipid metabolism is controlled by AMPK via suppression of fatty acid synthesis by directly phosphorylating acetyl CoA carboxylase (ACC1) and ACC2 with simultaneous induction of fatty acid oxidation (Figure 4). Furthermore, AMPK phosphorylates lipases such as ATGL and HSL (rate limiting enzyme in TG synthesis) to stimulate lipid absorption and release [169]. Similarly, the rate limiting enzyme in cholesterol biosynthesis, HMGCR (3-hydroxy-3-methyl-glutaryl-coA reductase) undergoes inhibitory phosphorylation by AMPK as a result of which the sterol and lipid synthesis within the cell is preprogrammed [170]. AMPK activation inhibits GPAT activity and subsequently TG synthesis. GPAT is an enzyme critical for the catalysis of TG synthesis [171]. Activation of AMPK elevated fatty acid oxidation and reduced hepatic lipid content in vivo [172]. Under conditions of excess nutrients, AMPK activation inhibits mTORC1 signaling and ER stress response to prevent hepatic lipid accumulation [173].

### 6.4. TFEB

Activation of transcription factor EB (TFEB) is another transcription mechanism that connects autophagy and lipid metabolism. TFEB, also known as a master regulator of lysosome biogenesis and autophagy, transcriptionally regulates lipid catabolism via PPAR1α and PPARgc1α [174]. Overexpression of TFEB perturbed the expression of genes involved in lipid metabolism [175] (Figure 4). Lipid droplets are the reliable energy reserves for the cells and are broken down under nutrient deficient conditions to meet the cellular energy demands. During such conditions, the lipid metabolism often tends to be dynamic and requires synergistic regulation of autophagy to maintain lipid flux. Several evidences suggest that TFEB translocates from cytoplasm to nucleus and regulates the lipophagy-related genes to modulate degradation and efflux of lipids [176].

### 6.5. TAK1

Participating in lipid metabolism and autophagy, TGFβ-activated kinase 1 (TAK1), remains yet another important member of the interactome connecting the two pathways. Similarly, TAK1 overexpression-induced autophagy was not cytoprotective but cytotoxic [177]. In contrast, TAK1-deficient hepatocytes display inhibited autophagy, expression of PPARα target genes and beta-oxidation with severe hepatosteatosis and high mTORC1 activity. The accumulation of lipids in TAK1- deleted livers under nutrient deprived conditions indicates the role of TAK1 in autophagy mediated clearance of lipids. TAK1 prevented excessive lipid accumulation through inhibition of mTORC1, activation of AMPK and subsequent induction of autophagy [178] (Figure 4). TAK1 additionally regulates energy expenditure, survival of adipocytes and high-fat-diet-induced obesity in mice [179].

### 6.6. NF-κB

Relish, among the five different DNA-binding subunits of NF-κB, governs the lipid metabolism during fasting conditions, conserving the cellular triglyceride level under metabolically adapted conditions in *Drosophila* by limiting the function of Foxo [180]. Activation of NF-κB was also reported to be a pathological mechanism in lipid metabolism and atherosclerosis [181]. During cardiac hypertrophy, NF-κB activation leads to a fall in fatty acid oxidation. Protein–protein interaction of the PPARβ/δ and P65 subunit of NF-κB was reported to be the underlying mechanism indicating the key role of NF-κB in lipid metabolism [182]. Furthermore, blocking NF-κB aids protection against insulin resistance and diet-induced hepatic steatosis [183]. (Figure 4).

## 7. The Lysosomal Lipid Handling in Macrophages

Lysosomes, as membrane-bound subcellular structures, play a pivotal role in regulating energy metabolism, nutrient sensing, recycling and degradation of extracellular materials, intracellular materials and in the export of recycled material via exocytosis [184,185]. Lysosomes play a predominant role in maintaining lipid homeostasis. The cellular trafficking pathways such as endocytosis and autophagy eventually converge at the lysosomes where both the exogenous and endogenous lipids are coordinated and sorted to various compartments [186,187]. LDL particles delivered into the lysosomal lumen are acted upon by lysosomal acid lipase type A (LIPA) to be de-esterified into cholesterol and triglyceride molecules and are transported out of the lysosomal lumen [186]. The lysosomal membrane proteins LAMP 1 and 2 have been reported to strongly bind to free cholesterol (FC) and facilitate their export from lysosomes [188]. Dysregulated lipid metabolism is often associated with lysosomal impairment. Correspondingly, impaired export of FC leads to their accumulation in the lysosomal lumen inhibiting the activity of Lysosomal acid lipase (LAL) and eventually that of lysosomes [189]. Further, lysosomes fail to maintain active pH when incubated with mildly OxLDL, AggLDL, and DISP for a longer period of time indicating the effect of cholesterol accumulation on LAL and lysosome activity [190]. Interestingly, formation of foam cells by arterial smooth muscle cells (SMCs) was reported to be affected by lysosome dysfunction. Macrophages express high level of LAL than arterial SMCs; in that, when treated with aggregated LDL (low-density lipoprotein), SMCs displayed accumulation and retention of neutral lipids in lysosomal compartments while most of them were stored as lipid droplets in the cytoplasm of macrophages [191]. In contrast, lysosomes of macrophage foam cells loaded with oxLDL were found to be accumulated with oxidized cholesterol esters and not free sterols. Lysosomal accumulation of cholesterol derived from mildly oxidized low density lipoprotein in lysosomes showed resistance for efflux [192]. Inhibition of lysosomal activity by chloroquine resulted in reduced AT lipolysis [193]. Additionally, lysosomal lipolysis is associated with macrophage polarization. In response to FFA, lysosomal lipolysis induces M2 phenotype in peritoneal and bone marrow-derived macrophages (BMDM) [194]. Lysosomes are recently identified as nutrient sensors. Further, their role in lipid catabolism and trafficking confirms the intimate link of lipid sensing and trafficking functions in lysosomes [186].

## 8. Conclusions

Lipid metabolism is an integral part of variety of functions in our body, including nerve impulse transmission, energy storage and hormone regulation. There are several studies that have reviewed lipid metabolism and associated diseases. Lipids play a pivotal role in the survival and functioning of macrophages. Lipids remain an integral part of macrophage functions such as phagocytosis and drive the macrophage polarization. Selective players such as AMPK and PPARα support macrophage survival via integration of pro-survival autophagy. While there are several lipid lowering drugs, such as statins, being identified and developed, their off-target effects remain a challenge for researchers. Although lipid accumulation/impaired lipid metabolism is widely reported to be one of the reasons for the pathogenesis of lipid metabolism-associated diseases, there is always a need for the development of effective modulators of lipid metabolism to study the underlying mechanism of disrupted lipid metabolism and uncontrolled accumulation of lipids in diseases.

## Figures and Tables

**Table 1:** Modulators of lipid metabolism.

SNo	Modulator	Mechanism	Effect
1	IFNγ [116]	ACAT1 mRNA expression	Lipid accumulation
2	Adipophilin [112]	Promotes TG and cholesterol storage	Increased cholesterol accumulation and reduced efflux
3	3-(5′-Hydroxymethyl-2′-furyl)-1-benzylindazole (YC-1) [113]	sGC/cGMP/PKG signaling pathway	Induces lipid accumulation
4	Lomitapide [117]	Inhibition of microsomal triglyceride transfer protein (MTP)	Lowers LDL cholesterol
5	Mipomersen [118]	Selective degradation of the apoB-100 mRNA-antisense oligonucleotide	Reduction in LDL-C and other lipoprotein levels
6	AAV8 (Adeno-associated viral serotype 8) TBG.hLDLR [119]	(AAV8)-low-density lipoprotein receptor (AAV8-LDLr) gene therapy	Reduction of plasma cholesterol levels
7	Inclisiran [120]	PCSK9 targeting siRNA	Decreased LDL-c levels
8	Bempedoic acid [121]	Inhibits ATP-citrate lyase	Lowers LDL-c in hypercholesterolaemia and established atherosclerosis
9	Gemcabene [122,123]	Down regulation of CRP (C-reactive protein)	Lipid lowering activity
10	Alipogene tiparvovec [124]	Adeno-associated viruses (AAVs) targeting lipoprotein lipase (LPL)- gene therapy	Reduce circulating plasma triglyceride levels
11	Pradigastat [125]	Specific inhibition of diacylglycerol acyltransferase 1 (DGAT1) blocking chylomicron triglyceride (TG) synthesis	Reduced TG levels in FCS (familial chylomicronemia syndrome)
12	Volanesorsen [126]	Antisense oligonucleotides targeting *ApoC*3 mRNA	Reducing triglyceride levels in patients with hypertriglyceridemia or familial chylomicronemia syndrome
13	Colesevelam HCl [127]	Accelerates cholesterol 7-α-hydroxylase mediated conversion of bile acids	Reduces total and low-density lipoprotein (LDL) cholesterol levels
14	Torcetrapib [114]	Inhibits cholesteryl ester-transfer protein (CETP)	Increased HDL-cholesterol and apolipoprotein A-I levels and decreased apolipoprotein B levels
15	Avasimibe [128]	Enhances cholesterol efflux and reduces LDL uptake	Reduces foam cell formation
16	Implitapide [129]	Inhibition of microsomal triglyceride transfer protein (MTP)	Reduction in TG levels and total cholesterol in plasma
17	Niacin [130]	Inhibits synthesis of apolipoprotein B-100 required for synthesis of VLDL	Increases HDL cholesterol levels and lowers LDL, VLDL and lipoprotein
18	Ezetimibe [131]	Reduces intestinal absorption of cholesterol	Reduces LDL cholesterol levels in patients with primary hypercholesterolemia
19	Cholestyramine [132]	Limits the reabsorption of bile acids in GI tract by forming a resin	Reduction in LDL level in primary hypercholesterolemia
20	Cholestipol [133]	Formation of complex with bile acids	Elimination of apoB-containing lipoproteins and LDL
21	Atorvastatin [134]	HMG-CoA reductase inhibition	Lowers blood total cholesterol and LDL
22	Fluvastatin [135]	Competitive inhibition of hydroxymethylglutaryl-coenzyme A (HMG-CoA) reductase	Decreases total cholesterol, LDL and triglyceride
23	Lovastatin [136]	HMG-CoA reductase inhibtion	Decreases triglyceride, VLDL and increases HDL
24	Pitavastatin sodium [137]	HMG-CoA reductase inhibtion	Decreases triglyceride, VLDL and increases HDL
25	Rosuvastatin [138]	Inhibition of HMG-CoA reductase	Lowers LDL-cholesterol, non-HDL cholesterol and total cholesterol
26	Simvastatin [139]	Competitive and reversible inhibition of HMG-CoA reductase enzyme	Reduced plasma LDL cholesterol levels
27	Pravastatin sodium [140]	HMG-CoA reductase inhibition	Reduces plasma LDL
28	Gernfibrosil [141]	Reduces TG production in liver	Reduction in plasma TG levels
